# Proximal Internal Iliac Artery Ligation in Obstetrics and Gynecology: An 11-Year Retrospective Single-Center Experience with No Clinically Detected Ischemic Complications

**DOI:** 10.3390/jcm15145658

**Published:** 2026-07-19

**Authors:** Stoyan Kostov, Yavor Kornovski, Stanislav Slavchev, Yonka Ivanova, Ekaterina Aleksandrova, Slavena Georgieva, Ilker Selcuk, Mohamed Wafa, Ihsan Hasan, Angel Yordanov, Nikolay Dimitrov, Rafał Watrowski

**Affiliations:** 1 Research Institute, Medical University Pleven, 5800 Pleven, Bulgaria; drstoqn.kostov@gmail.com; 2 Department of Gynecology, Hospital “Saint Anna”, 9002 Varna, Bulgaria; al3ksandr0va@abv.bg (E.A.); slavenageorgieva98@gmail.com (S.G.); 3 Department of Gynecology, Hospital “Saint Anna”, Medical University “Prof. Dr. Paraskev Stoyanov”, 9002 Varna, Bulgaria; ykornovski@abv.bg (Y.K.); st_slavchev@abv.bg (S.S.); yonka.ivanova@abv.bg (Y.I.); 4 Department of Gynecologic Oncology, Ankara Bilkent City Hospital, Maternity Hospital, Ankara 06800, Turkey; ilkerselcukmd@hotmail.com; 5 St. Louise Frauenklinik, 33098 Paderborn, Germany; m.wafa@vincenz.de; 6 University Specialized Hospital for Active Treatment in Oncology “Prof. Ivan Chernozemski”, 1756 Sofia, Bulgaria; ihsan_hasanov@abv.bg; 7 Department of Gynecologic Oncology, Medical University Pleven, 5800 Pleven, Bulgaria; angel.jordanov@gmail.com; 8 Department of Anatomy, Faculty of Medicine, Trakia University, 6000 Stara Zagora, Bulgaria; nikolay.dimitrov@trakia-uni.bg; 9 Department of Obstetrics and Gynecology, Helios Hospital Müllheim, 79379 Müllheim, Germany; 10 Faculty of Medicine, University of Freiburg, 79106 Freiburg, Germany

**Keywords:** internal iliac artery, bleeding, hemorrhage, hemostasis, ligation, gynecologic surgery, gynecologic oncology, placenta accreta, ischemic complications, buttock claudication, retrospective study

## Abstract

**Background:** Proximal ligation of the internal iliac artery (IIA) is a life-saving procedure for uncontrollable pelvic bleeding, often underutilized because of concerns about secondary buttock or pelvic organ ischemia, especially when ligation is performed proximal to the posterior IIA division. **Methods:** The primary aim was to evaluate clinically detected postoperative ischemic complications after IIA ligation, with secondary aims including technical success, primary hemostatic success, and overall hemorrhage control. In this single-center retrospective study, we evaluated 61 patients undergoing IIA ligation at a tertiary center in Bulgaria between January 2014 and March 2025. Demographics, indication, surgical approach, ligation level and laterality, comorbidities, and postoperative outcomes were analyzed. Ischemic complications were assessed by clinical examination and structured symptom inquiry at each follow-up visit (two visits in the first postoperative month, then at months 2, 3, 5, and 6). Imaging was reserved for patients with symptoms suggesting ischemia; none required further imaging. **Results:** IIA ligation was performed for obstetric (*n* = 18), benign gynecologic (*n* = 13), or gynecologic oncologic (*n* = 30) indications. The median age was 31.5 years (range 21–42), 50 years (range 35–76), and 64 years (range 31–81) in the obstetric, benign gynecologic, and gynecologic oncology groups, respectively. Bilateral ligation was achieved in 54/61 (88.5%) cases and unilateral ligation in 7/61 (11.5%). Bilateral proximal ligation was performed in 49/61 (80.3%); in 59/61 (96.7%) patients, at least one side was ligated proximally. Technical success was achieved in all patients (61/61, 100%). Primary hemostatic success was achieved in 59/61 (96.7%) patients. Hemostatic failure requiring escalation occurred in 2/61 (3.3%) patients: one with placenta accreta spectrum and one with placental abruption complicated by disseminated intravascular coagulation. Both required packing/laparostomy with delayed abdominal closure. Overall hemorrhage control was achieved in all patients (61/61, 100%). No clinical signs of ischemic complications (buttock, pelvic organ, spinal cord, or neuropathic) were recorded during the six-month follow-up period. **Conclusions:** In this cohort, proximal (including bilateral) IIA ligation was not associated with clinically detected ischemic complications and supported rapid hemorrhage control when performed by experienced surgeons. Larger prospective and comparative studies with standardized ischemia assessment are needed.

## 1. Introduction

Bleeding complications are among the most feared surgical complications and remain a leading cause of morbidity and mortality in gynecology and obstetrics (OB/GYN) [1,2,3]. A loss of approximately 30–40% of circulating blood volume typically results in cardiovascular instability, and losses exceeding 40% are usually immediately life-threatening [4,5]. Intraoperative or postoperative bleeding is not always predictable. Nevertheless, certain clinical scenarios are more frequently associated with life-threatening hemorrhage, such as uterine atony, placenta accreta spectrum (PAS), and peripartum trauma in obstetrics; radical cytoreductive procedures or lymphadenectomies in oncology; and surgery for very large myomas, retroperitoneal tumors or even sacrocolpopexy in benign gynecology [1,5,6,7]. Familiarity with pelvic surgical anatomy plays a critical role both in the occurrence and in the control of bleeding complications [1,8,9,10,11]. Similarly, perioperative morbidity and mortality decrease with increasing surgeon experience and hospital volume [12,13].

The internal iliac artery (IIA), historically referred to as the hypogastric artery, is the major arterial vessel in the pelvis. It contributes to the vascular supply of the pelvic viscera, pelvic sidewall, perineum and gluteal muscles. It can be divided either into anterior and posterior divisions or into parietal and visceral divisions. The posterior division of the IIA contains the following arteries: the superior gluteal artery, iliolumbar artery and lateral sacral arteries [8,14]. IIA ligation is a potentially lifesaving procedure used in obstetric and gynecologic surgery [15,16,17]. In obstetrics and gynecology, the procedure is particularly valuable in cases of severe pelvic hemorrhage, especially when endovascular interventions like arterial embolization or temporary balloon occlusion are not available [11,14,16]. Howard Kelly performed bilateral IIA ligation for a patient with locally advanced cervical cancer [18]. Despite early recognition of its “effectiveness, simplicity, and safety” in controlling severe pelvic hemorrhage in the landmark study of Siegel & Mengert from 1961 [19] and confirmation by more recent evaluations [20,21], the technique remains underutilized, mainly due to limited familiarity with the surgical approach and concerns about intraoperative and postoperative complications [11,15,22]. Only a few reports describe vascular or ischemic sequelae after unilateral or bilateral IIA ligation proximal to the posterior division [22,23,24,25,26].

The primary aim of the present retrospective single-center study was to evaluate postoperative ischemic complications after IIA ligation in an obstetric and gynecologic cohort, with a particular focus on proximal and bilateral ligation. Secondary aims were to describe technical success, primary hemostatic success, overall hemorrhage control, and the surgical circumstances in which IIA ligation was selected. The novel aspect of this study is the systematic assessment of ischemic outcomes after a high proportion of bilateral proximal IIA ligation within a cohort that includes both young obstetric patients and older oncologic patients with prevalent arterial comorbidities.

## 2. Materials and Methods

### 2.1. Study Design, Patient Selection, and Follow-Up

This single-center retrospective cohort study was conducted in the Department of Obstetrics and Gynecology at the “Saint Anna Hospital” in Varna, a tertiary care center for high-risk obstetrics and major gynecological procedures. Eligible patients were all women who underwent surgical IIA ligation between January 2014 and March 2025 for severe uterine, pelvic, or vaginal bleeding, or for planned/prophylactic vascular control in selected high-risk gynecologic procedures. Patients in whom hemorrhage was controlled by conservative, medical, or other surgical methods without IIA ligation were not eligible. Sixty-seven patients underwent IIA ligation during the study period.

The following data were recorded: age, gestational age in obstetric cases, diagnosis, comorbidities, pre- and postoperative hemoglobin levels, blood and plasma transfusions, type of ligation (transperitoneal/extraperitoneal, bilateral/unilateral, proximal or distal to the posterior division of the IIA), reinterventions, and perioperative complications. All procedures were performed by experienced gynecologic oncology surgeons (Y.K., S.K.) with sound knowledge of pelvic retroperitoneal anatomy. Informed consent for treatment and surgery was obtained during clinical care. Retrospective analysis of de-identified clinical data, including data from patients who died before follow-up completion, was covered by the institutional ethical approval. The Institutional Ethics Committee of the Saint Anna Hospital Varna approved the study (No. 1277-1/19 May 2026).

The primary outcomes were ischemic complications in the follow-up period of 6 months, and the secondary outcomes were technical success, hemostatic success, and overall hemorrhage control. We defined *technical success* as successful exposure and ligation of the intended IIA segment according to the planned laterality (unilateral/bilateral) and ligation level. *Primary clinical hemostatic success* was defined as control of pelvic/uterine bleeding after IIA ligation without escalation to an additional invasive hemostatic procedure during the early postoperative course; escalation procedures included pelvic packing (with scheduled re-laparotomy) or unscheduled re-laparotomy. Therapeutic ligation was performed in response to active bleeding. In planned or prophylactic cases, primary hemostatic success was defined as completion of the intended procedure without the need for additional invasive hemostatic escalation. *Overall hemorrhage control* was defined as bleeding ultimately controlled by the end of the hospitalization, with or without adjunct measures. *Re-bleeding* was defined as recurrent clinically relevant bleeding after initial control requiring re-intervention. *Ischemic complications* were defined as major ischemic events (e.g., buttock/gluteal necrosis, pelvic organ ischemia, spinal cord ischemia/neurologic deficit attributable to ischemia) and minor ischemic symptoms (e.g., transient buttock claudication or neuropathic symptoms), and were assessed during the hospitalization and the follow-up period of 6 months.

Postoperative follow-up consisted of two visits during the first postoperative month and additional visits at 2, 3, 5, and 6 months. Ischemic outcomes were assessed clinically during hospitalization and follow-up by a senior surgeon and a fellow who were not the primary operating surgeons responsible for the index procedure. The symptom inquiry followed a standardized checklist covering buttock pain or claudication, gluteal skin or soft-tissue changes, symptoms suggesting pelvic organ ischemia, urinary or bowel complaints, neurologic deficits, and neuropathic symptoms. Ultrasound was used as the first-line imaging modality when postoperative imaging was clinically indicated. MRI or CT was not performed for routine ischemia screening because no patient developed symptoms suggesting buttock, pelvic organ, spinal cord, or neuropathic ischemia.

**Figure 1:**
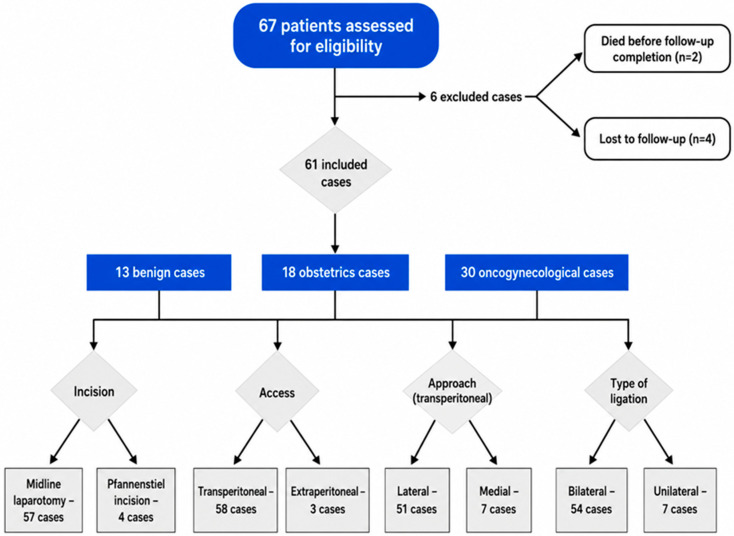
Patient assessment flowchart.

Patients were divided into three subgroups: group 1, obstetric cases; group 2, benign gynecologic surgery; and group 3, gynecologic oncology. Most patients (n = 57) underwent midline laparotomy; four obstetric patients underwent a Pfannenstiel incision. Most IIA ligations were transperitoneal (58/61). Two transperitoneal approaches were used: (1) a lateral approach through the posterior leaf of the broad ligament, parallel and lateral to the ovarian vessels, and (2) a medial approach parallel, medial, and caudal to the ovarian vessels [27]. The medial approach was used in seven obstetric patients when rapid access was required because of severe bleeding or pelvic sidewall adhesions. The lateral approach was used in the remaining transperitoneal cases because it allowed direct identification of the ureter and other retroperitoneal structures. Three patients with locally advanced cervical cancer and severe vaginal bleeding refractory to vaginal tamponade underwent extraperitoneal IIA ligation. No laparoscopic IIA ligation was performed in this study. Patients who underwent IIA ligation are summarized in Figure 1.

The decision to perform IIA ligation was made by the responsible senior surgeon, either intraoperatively or, in selected planned cases, before the main procedure, based on the clinical scenario, bleeding severity, available alternatives, and the anticipated speed of surgical vascular control. In obstetric cases, IIA ligation was used for catastrophic hemorrhage, most often before hysterectomy when hysterectomy was required, or after first-line measures such as uterine massage, uterotonics, uterine artery ligation, or tamponade were insufficient. In benign gynecology and gynecologic oncology, therapeutic IIA ligation was performed when severe pelvic, uterine, or vaginal bleeding could not be controlled by conventional hemostatic measures, including tranexamic acid and topical hemostatic agents. Planned or prophylactic ligation was used in selected high-risk cases, such as suspected uterine sarcoma or large retroperitoneal tumors. Extraperitoneal ligation was used as a palliative hemostatic procedure in women with advanced cervical cancer and persistent vaginal bleeding refractory to tamponade and hemostatic agents. Selective arterial embolization was not routinely available in our institution for obstetric or benign gynecologic emergencies, and no patient was referred from another institution for IIA ligation. In three cases with placenta previa and uterine atony, IIA ligation was performed after uterine artery ligation failed to control bleeding. In one case with uterine atony, IIA ligation was combined with Bakri balloon tamponade. B-Lynch sutures were not performed in patients with uterine atony. Hemorrhage control was confirmed by improvement in vital signs and visible reduction in pelvic, uterine, or vaginal bleeding. After adequate hemostasis with IIA ligation, no postoperative relaparotomy for rebleeding was required. Two patients with persistent bleeding after extraperitoneal ligation for advanced cervical cancer underwent definitive chemoradiation. Another patient with advanced cervical cancer underwent palliative radical hysterectomy because of severe bleeding and low hemoglobin levels, and was not sufficiently stable for transfer to radiology for palliative irradiation. In three women (one with cervical cancer, one with PAS, and one with placental abruption complicated by DIC), IIA ligation was combined with intra-abdominal packing; the packs were removed after 48 h.

Vasopressor support was used in 11 patients: five obstetric patients, two patients undergoing surgery for myoma, and four gynecologic oncology patients.

The level of ligation was selected according to the urgency of hemorrhage control, the surgical field, and the feasibility of safely identifying the IIA bifurcation. Proximal ligation, defined in this study as ligation at the IIA origin or within approximately 1–1.5 cm distal to the common iliac artery bifurcation, was preferred when rapid broad inflow reduction was needed or when distal dissection was unsafe because of hematoma, severe bleeding, adhesions, tumor involvement, or time pressure. During proximal ligation, the posterior division was not routinely exposed because ligation within 1–1.5 cm of the IIA origin will usually include the posterior division. The level of ligation was documented in operative notes and reviewed for this analysis. Distal ligation below the posterior division was considered only when the bifurcation was clearly exposed, and posterior-division preservation did not delay hemostasis. In unilateral or asymmetric bleeding, the contralateral side could be ligated at a different level according to the local operative situation. In emergencies, avoidance of prolonged dissection and avoidance of internal iliac vein injury had priority over formal posterior-division sparing.

### 2.2. Statistical Methods

Descriptive statistics were used to summarize patient, procedural, and outcome data. Continuous variables are reported as medians (range), and categorical variables as counts and percentages. Wilson 95% confidence intervals were calculated for key proportions using the score method without continuity correction [28], which provides accurate coverage for small samples and proportions near 0 or 1. No inferential comparisons between the obstetric, benign gynecologic, and gynecologic oncology subgroups were planned or performed because these groups represent clinically distinct indications and were not designed for comparative analysis. Statistical analyses were performed using R version 4.5.0 (R Foundation for Statistical Computing, Vienna, Austria).

### 2.3. Surgical Approaches and Technique

#### 2.3.1. Anatomy of the IIA

**Figure 2:**
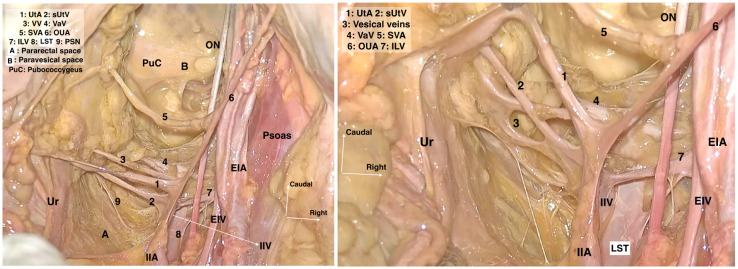
Anterior trunk branches of the right internal iliac artery and adjacent anatomy. The right image provides a closer view of the left image from a different angle to facilitate identification of the vascular branches. Branches of the internal iliac vessel system with the venous branches draining into the internal iliac vein, topographic orientation at the right pararectal and paravesical space. Cadaveric dissection by Ilker Selcuk. UtA: Uterine artery, sUtV: Superficial uterine vein, VV: Vesical vein, VaV: Vaginal vein, SVA: Superior vesical artery, OUA: Obliterated umbilical artery, ILV: Iliolumbar vein, IIV: Internal iliac vein, PSN: Pelvic splanchnic nerves, PuC: Pubococcygeus, LST: Lumbosacral trunk, Ur: Ureter, EIA: External iliac artery, EIV: External iliac vein, IIA: Internal iliac artery, ON: Obturator nerve.

**Figure 3:**
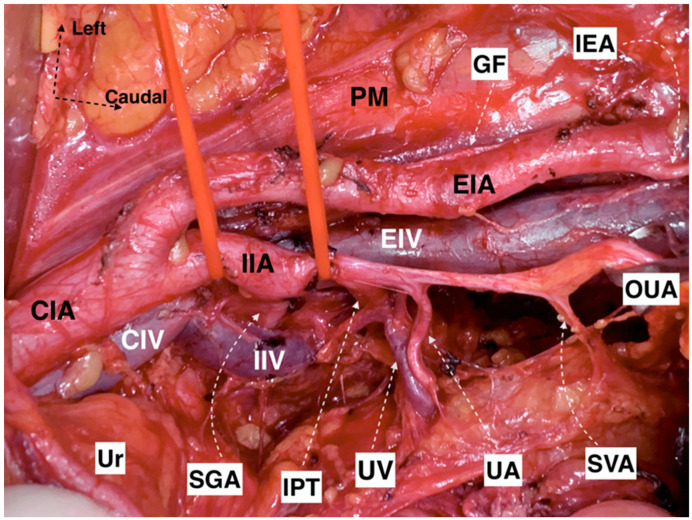
Internal iliac artery (IIA), posterior and anterior trunk. Surgical demonstration by Ilker Selcuk. IEA: Inferior epigastric artery, GF: Genitofemoral nerve, PM: Psoas major muscle, EIA: External iliac artery, EIV: External iliac vein, IIV: Internal iliac vein, CIA: Common iliac artery, CIV: Common iliac vein, Ur: Ureter, SGA: Superior gluteal artery, IPT: Ischiopudendal trunk, UV: Uterine vein, UA: Uterine artery, SVA: Superior vesical artery, OUA: Obliterated umbilical artery.

The anatomy of the IIA is described in detail in our previous study [14] and is summarized here. Generally, the bifurcation of the common iliac artery is at the level of the sacral promontory or at the level of the L5-S1 intervertebral disc. The anterior division of the IIA contains the following branches: umbilical artery, superior vesical artery, obturator artery, uterine artery, vaginal artery, middle rectal, internal pudendal and inferior gluteal artery. An inferior vesical artery may also be present in women, although its origin is variable. The posterior division contains the superior gluteal artery, the iliolumbar artery and the lateral sacral arteries. The posterior division usually arises from the dorsolateral aspect of the IIA and 4 cm below the origin of the IIA [14,29,30]. The anatomy of the IIA is demonstrated in Figure 2 and Figure 3.

Descriptions of pelvic arterial ligation differ according to the clinical setting and surgical approach (open versus laparoscopic) [14,27,31]; the descriptions and figures below show the IIA techniques used in this cohort.

#### 2.3.2. The Lateral Transperitoneal Approach

**Figure 4:**
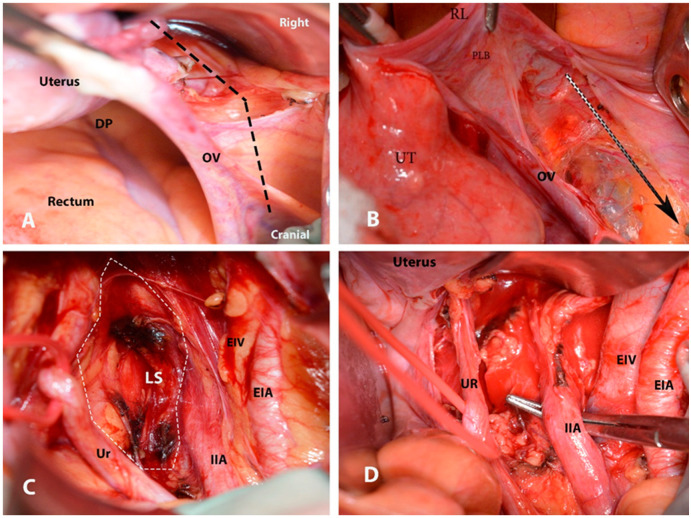
Intraperitoneal lateral approach to IIA ligation. Surgical demonstration, Stoyan Kostov, MD, PhD. (**A**) Incision line lateral to ovarian vessel. (**B**) Dissection of the fatty tissue, (**C**) Identification of the ureter, external and internal iliac vessels. Dissection of the lateral pararectal space. (**D**) Right-angle clap passes beneath the IIA. The tip of the clamp points at the adventitia of the artery; OV: ovarian vessels, DP: Douglas pouch, PLB: posterior leaf of broad ligament, UT: uterus, RL: round ligament, Ur: ureter, EIA: external iliac artery, EIV: external iliac vein, LS: Latzko’s pararectal space.

The posterior leaf of the broad ligament is incised parallel and lateral to the ovarian vessels (if the uterus is preserved), starting from the round ligament and continuing horizontally and cranially at the level of the common iliac artery; if the uterus has been removed, the pelvic peritoneum is incised lateral to the common iliac artery and extended caudally. The retroperitoneum is exposed after dissection of the areolar and adipose tissue. Tracking the caudal course of the common iliac artery, the external iliac artery and vein are identified. Ureter visualization is mandatory; the ureter is usually marked with a vessel loop. Dissection of the lateral pararectal (Latzko’s) space is not always necessary but provides additional access to the IIA in case of injury; it was omitted in obstetric cases to save time. Meticulous dissection between the IIA and internal iliac vein follows; dissecting the IIA adventitia helps find the correct plane. Complete mobilization is not required. The artery is gently elevated, and a right-angle clamp is passed beneath, from lateral to medial, with the tip pointing at the adventitia to prevent injury to the underlying vein. The clamp passes at the IIA origin or 1–1.5 cm caudally (proximal to the posterior division); when the posterior division is visible (mainly after pelvic lymphadenectomy), the clamp passes distal to it. In severe bleeding, proximal ligation is preferred, as distal ligation may be insufficient. One or two sutures are placed and tied firmly to avoid arterial transection; if two sutures are used, the artery is not cut between them. All retroperitoneal structures are re-checked before closure. Ipsilateral femoral artery pulsation is confirmed as the final step. The lateral approach is shown in Figure 4.

#### 2.3.3. The Medial Transperitoneal Approach

The medial transperitoneal approach was used mainly in obstetric cases because it provides rapid access to the iliac vessels. The posterior leaf of the broad ligament is incised medial and caudal to the ovarian vessels. In some cases, the peritoneal incision can be extended more caudally, and the peritoneum of the pouch of Douglas is incised. The ureter is identified either transperitoneally or by palpation with the thumb or index finger. The peritoneum is incised carefully, little by little, to avoid injury to the underlying ureter. The iliac vessels and the ureter are identified immediately after the peritoneal incision. The remaining surgical steps are similar to those used in the lateral transperitoneal approach.

#### 2.3.4. The Extraperitoneal Approach

**Figure 5:**
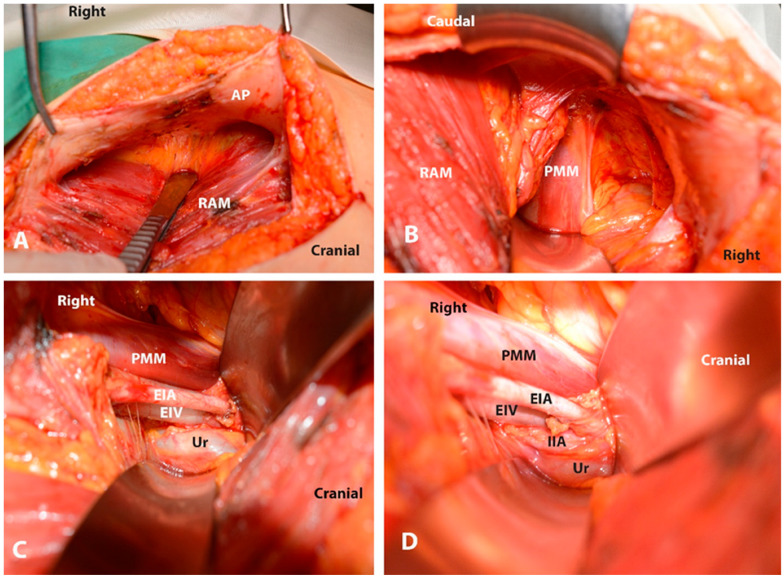
Extraperitoneal approach for IIA ligation. (**A**) Dissection between the rectus abdominis muscle and the aponeurosis; (**B**) Lateral and caudal dissection reveals the psoas major muscle; (**C**) Medial to the psoas major muscle dissection of the parietal peritoneum; Identification of the ureter, external iliac artery and external iliac vein; (**D**) Further cranial dissection shows the bifurcation of common iliac artery and the origin of IIA; RAM: rectus abdominis muscle, AP: aponeurosis of rectus abdominis muscle, PMM: psoas major muscle, EIA: external iliac artery, EIV: external iliac vein, Ur: ureter.

This access starts with a midline incision. The aponeurosis of the rectus abdominis muscle is incised, and the dissection proceeds between the muscle and its aponeurosis in a lateral direction. At the lateral edge of the muscle, the dissection continues in a dorsal direction until the identification of the psoas major muscle. The parietal peritoneum is retracted medially. Caudal and medial to the psoas major muscle dissection further identifies the ureter and the iliac vessels. The other steps of the procedure are similar to the other accesses. The extraperitoneal approach is shown in Figure 5.

## 3. Results

Among the 61 patients undergoing IIA ligation during the 11-year study period, 18 were obstetric cases, 13 were operated on for benign gynecologic pathologies, and 30 for gynecologic cancer. Bilateral IIA ligation was achieved in 54/61 (88.5%) cases, while 7/61 (11.5%) underwent unilateral ligation. Bilateral proximal ligation (proximal to the posterior division on both sides) was performed in 49/61 (80.3%). In addition, 3/61 (4.9%) patients had asymmetric bilateral ligation, with one side ligated proximal to the posterior division and the contralateral side ligated distal to it. Overall, in 59/61 (96.7%) patients, at least one pelvic side was ligated proximally.

Across the three subgroups, technical success was achieved in all patients (61/61, 100%). Primary clinical hemostatic success was observed in 16/18 (88.9%) obstetric, 13/13 (100%) benign gynecologic, and 30/30 (100%) gynecologic oncology cases (overall 59/61, 96.7%). Hemostatic failure requiring escalation occurred in two obstetric patients, one with PAS and one with placental abruption complicated by DIC. Both were managed with damage-control surgery, abdominal packing/laparostomy, and delayed abdominal closure. Overall hemorrhage control was achieved in all patients (61/61, 100%). No re-bleeding requiring re-intervention and no major or minor clinically detectable ischemic complications were recorded in any subgroup. Two intraoperative internal iliac vein lesions occurred. One lesion arose during IIA dissection in a benign gynecologic case and was the only vascular complication directly related to IIA ligation; it was controlled with a hemostatic sponge without additional suturing. The second lesion occurred during pelvic lymph node dissection in an oncologic patient and was related to that part of the procedure, not to the IIA ligation. Persistent or prolonged bleeding after IIA ligation was recorded in five patients: one obstetric patient who required packing/laparostomy and four oncologic patients managed without surgical re-intervention. A second obstetric patient with placental abruption complicated by DIC required packing/laparostomy as part of damage-control surgery. Other perioperative complications included two bladder injuries, three ureterovaginal fistulas, one rectovaginal fistula, and one pulmonary embolism. These events are described in the corresponding subgroup sections. A summary of patient characteristics and outcomes is provided in Table 1. Patient-level hemoglobin values, transfusion requirements, and reinterventions are provided in Table A1, Table A2 and Table A3.

Six of the 67 patients who underwent IIA ligation were not included in the final six-month ischemic-outcome analysis. Two patients died before follow-up completion. One obstetric patient had intrapartum amniotic-fluid embolism, uterine atony, disseminated intravascular coagulation (DIC), hysterectomy after bilateral proximal IIA ligation, and subsequent death in the intensive care unit. The second patient had pelvic sidewall recurrence of cervical cancer, uncontrollable intraoperative bleeding, DIC, bilateral proximal IIA ligation, and subsequent death in the intensive care unit. Four additional patients were lost to follow-up (two with cervical cancer, one with uterine sarcoma, and one with ovarian cancer). Among these four patients, three underwent transperitoneal therapeutic bilateral proximal ligation during total abdominal hysterectomy with bilateral salpingo-oophorectomy, and one underwent extraperitoneal palliative bilateral proximal ligation as the only surgical procedure for advanced cervical cancer.

All 61 patients included in the final analysis completed a six-month clinical follow-up. No patient reported buttock claudication, gluteal pain with walking, gluteal skin or soft-tissue ischemia, new pelvic organ ischemia symptoms, spinal cord ischemia, or neuropathic symptoms attributable to IIA ligation. No clinically detected ischemic complications were observed among patients who completed six months of follow-up. The corresponding Wilson 95% confidence interval was 0–5.9%. No postoperative imaging for suspected ischemia was required because no patient developed symptoms warranting further evaluation.

### 3.1. IIA Ligation in Obstetrics

The median age of patients was 31.5 years (range 21–42). Indications for IIA ligation in obstetrics were placenta previa (n = 5, 27.8%), PAS (n = 5, 27.8%), uterine hematoma combined with uterine atony (n = 3, 16.7%), uterine atony (n = 2, 11.1%), cesarean scar hematoma (n = 1, 5.6%), molar pregnancy (n = 1, 5.6%), and placental abruption with DIC (n = 1, 5.6%). All obstetric patients underwent therapeutic IIA ligation for severe bleeding. As described above, IIA ligation was performed before hysterectomy in all cases in which the uterus was removed. Three patients had preeclampsia, and one had transient acute kidney injury. Uterine preservation was achieved in 11 patients (61.1%); among these, placenta previa accounted for almost half of the cases. In all 18 obstetric cases, IIA ligation was bilateral and performed proximal to the posterior division. In two patients, hemostasis required abdominal packing/laparostomy as part of a damage-control approach: one had persistent bleeding associated with PAS, and the other had placental abruption complicated by DIC. One patient developed severe vaginal bleeding during medical abortion for molar pregnancy and underwent sectio parva followed by bilateral IIA ligation. Two PAS patients sustained bladder injury during hysterectomy.

In the obstetrics subgroup, 16/18 (88.9%) patients received transfusions of blood products. The median Hb drop (the difference between pre- and postoperative Hb) was 0.8 g/dL (range −1.7 to 5.4 g/dL), where a negative value indicates postoperative Hb increase. No perioperative complications attributable to IIA ligation were observed, and no clinically detectable ischemic complications were recorded. Individual obstetric cases are shown in Table 2.

### 3.2. IIA Ligation for Benign Gynecological Pathologies

The median age of patients was 50 years (range 35–76). Indications for IIA ligation in benign gynecology were huge uterine myomas (mainly retroperitoneal; n = 9, 69.2%), retroperitoneal ovarian cysts (n = 2, 15.4%), parametritis extending to the pelvic sidewall (n = 1, 7.7%), and retroperitoneal hematoma after laparoscopic sacrocolpopexy (n = 1, 7.7%). In 9/13 (69.2%) patients, ligation was bilateral and performed proximal to the posterior division; in the remaining 4/13 (30.8%) patients, ligation was unilateral. In four cases of retroperitoneal myoma, the artery was ligated before hysterectomy. In one fertility-preserving case with a huge uterine myoma, IIA ligation reduced bleeding sufficiently to avoid hysterectomy and preserve the uterus. In three patients, IIA ligation was performed during relaparotomy for postoperative bleeding (two after hysterectomy and one after laparoscopic sacrocolpopexy); one of these patients developed pulmonary embolism after the index operation, and another had a history of aortic prosthesis with anticoagulant treatment. Most benign gynecologic patients underwent therapeutic IIA ligation. Three women underwent planned or pre-emptive ligation: two with large retroperitoneal myomas and one with myoma/ovarian cyst who required relaparotomy for bleeding shortly after pulmonary embolism; in the latter case, extensive retroperitoneal hematoma led to IIA ligation before definitive hemostasis. One intraoperative complication during IIA ligation was recorded: a small lesion of the ipsilateral internal iliac vein, which was controlled with a hemostatic sponge without the need for additional sutures.

In this subgroup, 8/13 (61.5%) patients received transfusions of blood products. The median Hb drop was 2.0 g/dL (range −1.5 to 5.8 g/dL). No postoperative complications attributable to IIA ligation were recorded. Arterial hypertension (AHT) was the most common comorbidity and was present in 8 women (61.5%). Individual benign gynecologic cases are shown in Table 3.

### 3.3. IIA Ligation for Gynecological Cancer

The median age of patients was 64 years (range 31–81). Indications for IIA ligation in gynecologic oncology were cervical cancer (n = 14, 46.7%), uterine sarcoma (n = 6, 20.0%), ovarian cancer (n = 7, 23.3%), endometrial carcinosarcoma (n = 2, 6.7%), and vaginal cancer (n = 1, 3.3%). Of the cervical cancer cases, three patients had advanced disease and underwent extraperitoneal palliative ligation as the only procedure for hemorrhage control. In four women (13.3%) with uterine sarcoma, the artery was ligated before hysterectomy. Unilateral IIA ligation was performed in 3/30 (10.0%) cases. Bilateral proximal ligation was performed in 22/30 (73.3%), while bilateral distal ligation was performed in 2/30 (6.7%). Asymmetric bilateral ligation (one side proximal and the other distal) was recorded in 3/30 (10.0%) and was typically used when the posterior division was identified during pelvic lymph node dissection or during extended pelvic sidewall dissection in less severe bleeding. Planned or prophylactic ligation was performed in three oncologic patients: two women with uterine sarcoma and one young woman with ovarian cancer and frozen pelvis. In the two uterine sarcoma cases, tumor extension toward the right pelvic sidewall involved branches of the IIA, and unilateral ligation was selected. AHT was the most common comorbidity (n = 19, 63.3%), followed by diabetes mellitus (n = 5, 16.7%).

In the oncologic subgroup, 13/30 (43.3%) patients received transfusions of blood products. The median Hb drop was 2.5 g/dL (range −4.7 to 6.6 g/dL). No intraoperative or postoperative ischemic complications associated with IIA ligation were observed. In three patients with advanced cervical cancer undergoing extraperitoneal ligation, bleeding persisted or was prolonged but did not require surgical re-intervention. Three patients developed postoperative ureterovaginal fistulas after radical hysterectomy for cervical cancer. In another patient, an internal iliac vein lesion occurred during pelvic lymph node dissection; this complication was related to the lymphadenectomy and not to the IIA ligation. Individual gynecologic oncology cases are summarized in Table 4.

## 4. Discussion

Fifty-six years after the landmark evaluation of 200 patients undergoing IIA ligation by Burchell and Mengert, their statement remains pertinent: “IIA ligation is a life-saving procedure, if employed promptly. On the other hand, it remains unused and, too often, unknown by the average practicing obstetrician-gynecologist. If he knows about it, he is loath to perform it for the first time on a seriously ill patient in an emergency situation” [15]. In experienced hands, ligation of the IIA can be completed in less than 5 min [32]. After ligation, pulse pressure and mean pressure in the ipsilateral and contralateral pelvis significantly decrease, converting the arterial system into a low-pressure venous-like system; consequently, the “trip hammer effect” of arterial pulsation disappears [11,33,34,35]. The subsequent preservation of pelvic organ function is explained by collateral recruitment. Over time, collateral circulation restores perfusion, with the deep femoral artery often serving as a principal source of revascularization through anastomotic pathways such as the medial femoral circumflex-obturator artery and the lateral femoral circumflex-superior gluteal artery connections [11,35]. However, the specific collateral routes may differ between individuals. In the arteriographic study of Iwata et al., for example, collateral supply involved a rectal branch of the inferior mesenteric artery, the superior gluteal artery, and the inferior epigastric artery [36]. Uterine perfusion is maintained via the ovarian artery and additional collaterals [11,35,36,37,38], and available clinical data indicate that IIA ligation does not impair future fertility [39,40]. Although IIA ligation continues to be part of guidelines and expert reviews for uncontrolled intra- or postpartum hemorrhage [41,42,43], it is still not widely practiced by obstetric and gynecologic surgeons [11,14,44].

In our cohort, the procedure was technically feasible in all patients, and the overall clinical outcome was favorable even in scenarios that are commonly perceived as “high risk” (older gynecologic oncology patients and a high proportion of bilateral proximal ligation). The two hemostatic failures arose from different bleeding mechanisms. One occurred in PAS, where extensive collateral circulation may limit the effect of IIA ligation, and the other followed placental abruption complicated by DIC, in which coagulopathic bleeding cannot be controlled by arterial inflow reduction alone. Both patients required packing/laparostomy and delayed abdominal closure as part of damage-control surgery. Importantly, overall hemorrhage control was ultimately achieved in all patients, and no re-bleeding requiring re-intervention occurred. In this sense, our results support a pragmatic view of IIA ligation as a rapid and effective inflow-reduction maneuver across many surgical scenarios, but it does not eliminate the multimodal approach when the bleeding mechanism is complex (particularly in PAS) [45].

From a practical standpoint, the underuse of IIA ligation has several causes. Modern advances in imaging, together with the increasing availability of selective arterial embolization (SAE) and temporary balloon occlusion (TBO), have shifted attention away from surgical vascular control [20,41,43,46,47,48,49]. These endovascular approaches are valuable in centers where experienced interventional radiology teams are immediately available, especially for planned high-risk cases. In unstable or unexpected intraoperative hemorrhage, however, transfer, setup, and procedural preparation may delay hemostasis, and caesarean delivery or hemodynamic shock has been associated with high failure rates in embolization series [50,51]. A second cause of underuse is limited familiarity with pelvic retroperitoneal anatomy and limited hands-on training for this specific dissection [10,52,53]. Retroperitoneal exposure and pelvic sidewall dissection are more routinely performed by gynecologic oncologists than by many obstetricians, which may explain why gynecologic oncologists are often involved in PAS surgery and other difficult hemorrhage scenarios [54,55,56]. Third, concerns about postoperative ischemic complications to the buttocks and pelvic viscera, especially after proximal ligation, are largely extrapolated from non-gynecologic literature and only rarely reported in gynecology [22,23,57,58].

A key question is what “proximal to the posterior division” implies anatomically and how reliably the posterior division can be spared in emergency conditions. Cadaveric and imaging studies indicate that the distance from the IIA origin to the bifurcation into anterior and posterior divisions varies widely. In 54 female cadavers, Bleich et al. reported that the posterior division will be spared if the ligation is placed approximately 30 mm after the origin of IIA, and calculated that the posterior branch can be spared in about 95% of cases when the distance is about 50 mm [29]. Cadaveric data from Terek et al. similarly demonstrated mean distances of 40.2 mm (right) and 38.4 mm (left) from IIA origin to the posterior division branching point [30]. CT angiography confirms both the range and the variability: Hajdyła et al. found a median distance of 42.48 mm (IQR 37.17–52.81; range 20.27–84.94 mm) from the IIA origin to the bifurcation into anterior and posterior divisions [59]. Importantly, “posterior-division sparing” as a binary concept is not fully correct anatomically, as pelvic branching patterns are heterogeneous; for example, in CT angiographies, at least one artery typically regarded as an anterior-division branch originated from the posterior division in 13.5% of pelvic sides [59]. Against this anatomical background-and considering that the IIA trunk can be short (mean 3.82 cm, range 1.28–8.13 cm) and shows population-level variation in bifurcation patterns [60,61]-the proximal ligation of the IIA in our cohort (ligation at the IIA origin or within 1–1.5 cm distal to the common iliac artery bifurcation) includes the posterior division in the vast majority of cases.

The concerns about ischemic complications often arise from non-gynecologic literature and should be interpreted with caution. Reports on severe ischemic sequelae after IIA ligation typically involve patients with multiple comorbidities or previous pelvic interventions. Andriole and Sugarbaker described bladder, vaginal, perineal, and buttock necrosis in a patient with longstanding diabetes, hypertension, and prior pelvic radiation for rectal carcinoma [22]; Asgari et al. reported gluteal necrosis and lumbosacral plexopathy following renal transplantation with ipsilateral IIA ligation in a male patient with type 1 diabetes, hypertension, and opium addiction [57]. A single case of peripheral nerve ischemia after bilateral IIA ligation in an obstetric patient with preeclampsia has been attributed to preeclampsia-induced endothelial dysfunction [23]. Smith et al. reported an 11-cm unilateral buttock necrosis after uterine artery embolization during conservative management of placenta percreta [58]. Severe ischemic complications were observed more commonly after aortoiliac reconstruction affecting IIA: an older case series of eight patients undergoing aortoiliac bypass reported paralysis in all eight patients, buttock necrosis in four patients, anal and bladder sphincteric dysfunction in two patients, colorectal ischemia in three patients, with a 63% (5/8) mortality [62]. Additionally, Picone et al. described spinal cord ischemia following abdominal aortic operations [63]. On the contrary, a meta-analysis in the context of endovascular abdominal aortic aneurysm repair found that major ischemic complications (bowel, gluteal, spinal cord) were very rare, while buttock claudication was substantially more frequent (in ca. 28% of cases) [64]. Consequently, ischemic risk appears to be driven primarily by the patient’s baseline vascular status and the extent of concurrent arterial manipulation, not by IIA ligation itself.

In contrast to the relatively low complication rates from IIA ligation, ischemic complications following IIA embolization are more frequently reported [65,66,67], and complication rates do not appear to differ substantially by embolic material [68]. A systematic review comparing IIA embolization and ligation found a significantly higher rate of ischemic complications after embolization (16.2%) compared with ligation (3.9%), with buttock claudication being the most frequent event (5.2%, mostly reversible); buttock necrosis (1.4%) and spinal cord ischemia (1.9%) were rare in both groups, and no cases of colon ischemia were reported [68]. One plausible explanation is that embolization may increase the risk of distal atheroembolism or may occlude distal vessels more extensively than surgical ligation, depending on catheter position and embolic distribution [68]. The same review found no difference in complication rates between bilateral and unilateral ligation, and the incidence of ischemic complications after IIA ligation in obstetrics and gynecology was lower than in vascular surgery, oncology, or trauma patients [68]. These data are consistent with our observation of no clinically apparent ischemic complications despite a high proportion of bilateral proximal ligation, including in patients with comorbidities.

Comparisons between IIA ligation in obstetric or gynecologic hemorrhage and its use in other disciplines require caution. In traumatic pelvic fracture bleeding, bilateral IIA ligation has mainly been used in profoundly unstable patients when angiography was unavailable or transfer could not be performed safely. The evidence from the trauma literature is observational and heterogeneous; however, ischemic sequelae were uncommon among reported survivors, including patients treated with bilateral IIA ligation and pelvic packing as part of damage-control surgery [69,70].

Several risk factors have been linked to ischemic complications after IIA ligation or embolization, and they largely converge on impaired collateral function or additional vascular injury [22,23,63,68]. Older age, hypertension, and diabetes mellitus may reflect atherosclerotic compromise of collaterals or endothelial dysfunction; preeclampsia is associated with endothelial dysfunction; prior chemotherapy or radiotherapy can cause endothelial damage and radiation-induced vasculopathy; cancer patients may have hypercoagulability; vascular patients often have atherosclerotic occlusion; and left colon resection (via interruption of the inferior mesenteric or superior rectal arteries) may reduce anastomotic pathways between these vessels and the IIA [22,23,63,68].

In our cohort, no patient developed major or minor clinically detected ischemic complications during the six-month follow-up period, despite a high prevalence of recognized vascular risk factors. Arterial hypertension was present in 27/61 patients overall (44.3%). Among the 43 patients in the benign gynecologic and oncologic groups, 27 (62.8%) had arterial hypertension, six (14.0%) had diabetes mellitus, and five had both conditions. Two patients had both heart failure and hypertension and underwent bilateral proximal ligation. One patient underwent hysterectomy with pelvic lymph node dissection after definitive chemoradiation for advanced cervical cancer and residual tumor, combined with bilateral proximal IIA ligation and abdominal packing. Three patients developed ureterovaginal fistulas after radical hysterectomy for cervical cancer, and one obstetric patient with PAS developed a rectovaginal fistula after hysterectomy and damage-control packing. These fistulas were attributed to the underlying disease and the main surgical procedures, not to IIA ligation. The low incidence of clinically detected ischemic events in obstetrics and gynecology is biologically plausible because of the dense collateral network between IIA branches and the external iliac, abdominal, and femoral arterial systems. A detailed description of these collaterals has been provided in our previous work on IIA ligation [14].

Clinical series in obstetrics and gynecology generally report few clinically evident ischemic events after IIA ligation [21,39,43,71,72,73,74,75]. The main operative question concerns the hemostatic effect of the ligation level in different bleeding patterns. During emergency surgery, additional dissection to identify and preserve the posterior division may delay hemorrhage control and increase the risk of internal iliac vein injury [21]. Distal ligation may also leave collateral flow through lower vaginal, inferior vesical, and other extrauterine pathways intact [37,46]. This anatomy may explain why comparative studies in placenta previa/PAS have not shown a consistent benefit of IIA ligation. In the recent meta-analysis by El-Sherbiny et al., the procedure was not associated with significant reductions in blood loss, transfusion, operative time, postoperative hemoglobin decrease, bladder injury, or hospital stay, although several outcomes showed substantial heterogeneity [36,45,46,76]. In severe placenta previa/percreta, aortic balloon occlusion was associated with lower blood loss and shorter surgery than IIA ligation, while IIA ligation remained an available option when endovascular support could not be provided [77]. In a comparative peripartum hysterectomy cohort, Zahoor et al. reported numerically more ICU admissions and maternal deaths among patients managed without bilateral IIA ligation and advocated earlier use of the procedure in massive postpartum hemorrhage [78]. IIA ligation therefore appears most useful as a rapid surgical means of reducing pelvic arterial inflow during emergency bleeding, in hospitals without immediate endovascular support, or within a stepwise hemorrhage-control strategy. Extensive collateralization in PAS may still necessitate hysterectomy, packing, endovascular intervention, or damage-control surgery.

In our cohort, proximal IIA ligation promptly reduced pelvic inflow across benign, obstetric, and oncologic indications and avoided additional dissection of the posterior division during emergency surgery. In two obstetric cases, hemorrhage could not be controlled by bilateral proximal ligation and hysterectomy alone, and packing/laparostomy with delayed abdominal closure was required. In the remaining patients, proximal IIA ligation contributed to prompt hemorrhage reduction, and bleeding was ultimately controlled in all cases.

A particular strength of our study is that it reports outcomes in a cohort with a high proportion of IIA ligations performed proximal to the posterior division and often bilaterally, an approach that is frequently avoided because of fear of ischemic complications. Another strength is the inclusion of distinct clinical categories (obstetrics, benign gynecology, and gynecologic oncology), with a substantial proportion of patients carrying comorbidities that, together with proximal ligation, have been described as risk factors for ischemic complications. Despite this, no patient developed a clinically detected ischemic complication during the six-month follow-up.

The limitations of this study include its retrospective design, single-center setting, modest sample size, and absence of a control or comparator group. The cohort combines obstetric, benign gynecologic, and oncologic indications with markedly different baseline characteristics, bleeding mechanisms, and operative circumstances. The pooled estimates describe experience with a shared surgical intervention in a clinically heterogeneous population. The choice of IIA ligation, the timing of ligation, and the selection of proximal versus distal ligation were determined by clinical urgency, surgical anatomy, surgeon expertise, and local availability of alternatives; this introduces selection bias and limits direct comparison with distal ligation, embolization, or balloon occlusion. Because the cohort included 61 evaluable patients, rare ischemic events cannot be ruled out statistically. With zero observed events, the upper 95% confidence limit for clinically detectable ischemic complications is 5.9%; consequently, the study is underpowered to detect complication rates below this threshold. Ischemic outcomes were assessed clinically during hospitalization and scheduled follow-up, without a validated ischemia-specific scale and without routine postoperative vascular imaging. The study therefore may miss subclinical ischemia, very mild transient claudication, or symptoms not reported by patients. Follow-up of six months is adequate for acute and subacute ischemic events, but does not address very long-term sequelae. Two patients who died from DIC (one after amniotic-fluid embolism) and four patients lost to follow-up were excluded from the final six-month ischemic-outcome analysis; had they survived and completed follow-up, the primary outcome estimate could have differed. Further prospective multicenter studies with standardized postoperative ischemia assessment are needed to compare the safety and efficacy of IIA ligation, balloon occlusion, and embolization in comparable cohorts, particularly in settings where the choice of strategy is not predetermined by personal or logistic constraints. Future work should also address potential long-term consequences of therapeutic IIA occlusion that have so far received limited attention, such as urinary or fecal continence [79].

## 5. Conclusions

In this retrospective single-center series of 61 obstetric, benign gynecologic, and gynecologic oncology patients with completed six-month follow-up, IIA ligation achieved technical success in all cases and supported hemorrhage control across indications despite frequent bilateral proximal ligation (80.3%) and relevant vascular comorbidities. Primary hemostatic success was achieved in 59/61 (96.7%). Escalation with packing/laparostomy was required in 2/61 (3.3%) obstetric patients, one with PAS and one with placental abruption complicated by DIC. Overall hemorrhage control was ultimately achieved in all patients, and no re-bleeding requiring re-intervention occurred. No buttock, pelvic organ, spinal cord, or neuropathic ischemic complications were clinically detected during follow-up. Within the limitations of the study design-particularly the modest sample size, lack of routine vascular imaging or a validated ischemia assessment instrument, exclusion of six patients from the final ischemic-outcome analysis, and upper 95% confidence limit of 5.9% for clinically detectable ischemic complications-proximal (including bilateral) IIA ligation was not associated with clinically detected ischemic complications in this cohort. It may serve as a rapid inflow-reduction option in experienced hands when immediate surgical control is required or endovascular support is unavailable. Larger prospective studies with standardized postoperative assessment are needed to define safety and comparative effectiveness versus endovascular strategies.

## Figures and Tables

**Table 1:** Summary of patient characteristics and outcomes by indication group.

Measure	Overall (n = 61)	Obstetrics (n = 18)	Benign Gyn (n = 13)	Gyn Onco (n = 30)
Age, years-median (range)	50 (21–81)	31.5 (21–42)	50 (35–76)	64 (31–81)
Bilateral IIA ligation, n (%)	54 (88.5%)	18 (100%)	9 (69.2%)	27 (90.0%)
Unilateral IIA ligation, n (%)	7 (11.5%)	0 (0%)	4 (30.8%)	3 (10.0%)
Proximal to PD-both sides, n (%)	49 (80.3%)	18 (100%)	9 (69.2%)	22 (73.3%)
Proximal to PD- ≥ 1 side, n (%)	59 (96.7%)	18 (100%)	13 (100%)	28 (93.3%)
Technical success, n (%) [95% CI]	61 (100%) [94.1–100%]	18 (100%) [82.4–100%]	13 (100%) [77.2–100%]	30 (100%) [88.6–100%]
Overall hemorrhage control, n (%) [95% CI]	61 (100%) [94.1–100%]	18 (100%) [82.4–100%]	13 (100%) [77.2–100%]	30 (100%) [88.6–100%]
Re-bleeding requiring re-intervention, n (%) [95% CI]	0 (0%) [0–5.9%]	0 (0%) [0–17.6%]	0 (0%) [0–22.8%]	0 (0%) [0–11.4%]
Primary clinical hemostatic success, n (%) [95% CI]	59 (96.7%) [88.8–99.1%]	16 (88.9%) [67.2–96.9%]	13 (100%) [77.2–100%]	30 (100.0%) [88.6–100.0%]
Hemostatic failure requiring escalation, n (%) [95% CI]	2 (3.3%) [0.9–11.2%]	2 (11.1%) [3.1–32.8%]	0 (0%) [0–22.8%]	0 (0.0%) [0.0–11.4%]
Median preop. Hb, g/dL (range)	11.0 (4.0–16.7)	10.0 (7.7–12.8)	12.6 (8.2–16.7)	11.5 (4.0–15.5)
Median postop. Hb, g/dL (range)	9.0 (6.1–13.5)	9.0 (6.1–11.0)	9.0 (6.1–13.5)	9.0 (7.0–13.0)
Median Hb drop *, g/dL (range)	1.9 (−4.7 to 6.6)	0.8 (−1.7 to 5.4)	2.0 (−1.5 to 5.8)	2.5 (−4.7 to 6.6)
Patients transfused, n (%) [95% CI]	37 (60.7%) [48.1–71.9%]	16 (88.9%) [67.2–96.9%]	8 (61.5%) [35.5–82.3%]	13 (43.3%) [27.4–60.8%]
Ischemic complications (major/minor), n (%) [95% CI]	0 (0%) [0–5.9%]	0 (0%) [0–17.6%]	0 (0%) [0–22.8%]	0 (0%) [0–11.4%]

95% confidence intervals were calculated using the Wilson score method without continuity correction. * Hb drop was calculated as preoperative Hb minus postoperative Hb; negative values indicate a higher postoperative than preoperative Hb level. Abbreviations: preop, preoperative; postop, postoperative; PD, posterior division.

**Table 2:** History of patients who underwent internal iliac artery ligation in the obstetric group.

Case No.	Age	WoG/ Delivery Mode	Comorbidities	Obstetrics Pathology	Ligation Before/After Hysterectomy vs. Uterine Preservation	Type of Ligation	Perioperative Complications
1	21	18/MA/SP	Hypoproteinemia, Transient AKI	Molar pregnancy	Uterine preservation	Bilateral/ Above the PD	No
2	36	36/CS	No	PAS	Before/Abdominal packing	Bilateral/ Above the PD	Persistent bleeding; Rectovaginal fistula; bladder injury
3	25	37/CS	No	Placenta previa	Uterine preservation	Bilateral/ Above the PD	No
4	33	36/CS	No	PAS	Before	Bilateral/ Above the PD	Bladder injury
5	32	36/CS	No	Placenta previa	Uterine preservation	Bilateral/ Above the PD	No
6	30	37/CS	No	Placenta previa	Uterine preservation	Bilateral/ Above the PD	No
7	42	34/CS	Hashimoto thyroiditis	Placenta previa	Uterine preservation	Bilateral/ Above the PD	No
8	32	36/CS	No	PAS	Before	Bilateral/ Above the PD	No
9	36	37/CS	Preeclampsia/ Varicosis cruris	PAS	Before	Bilateral/ Above the PD	No
10	31	27/CS	Preeclampsia/anemia	Cesarean Scar hematoma	Uterine preservation	Bilateral/ Above the PD	No
11	29	37/CS	No	Placenta previa	Uterine preservation	Bilateral/ Above the PD	No
12	32	39/CS	Myopia/ Astigmatism	Uterine atony	Uterine preservation	Bilateral/ Above the PD	No
13	32	36/CS	Preeclampsia	Placental abruption/ DIC syndrome	Before/ Abdominal packing	Bilateral/ Above the PD	No
14	31	38/CS	No	Unilateral RPH/Atony	Before	Bilateral Above the PD	No
15	34	38/CS	No	Unilateral RPH /Atony	Uterine preservation	Bilateral Above the PD	No
16	23	38/CS	No	Unilateral RPH	Uterine preservation	Bilateral/ Above the PD	No
17	29	38/CS	No	RPH after SC/Atony	Uterine preservation	Bilateral/ Above the PD	No
18	31	36/CS	No	PAS	Before	Bilateral/ Above the PD	No

WoG—week of gestation; PD—posterior division; PAS—Placenta accreta spectrum; AKI—acute kidney injury; CS—Cesarean section; MA—medical abortion; SP—sectio parva; RPH—retroperitoneal hematoma.

**Table 3:** History of patients who underwent internal iliac artery ligation in gynecological cases.

No	Patient’s Age	Comorbidities	Diagnosis	Procedure	Type of Ligation	Perioperative Complications	Type of Procedure
1	42	No	Uterine myoma	TAH without BSO	Bilateral/proximal *	No	Therapeutic
2	50	AHT class II	Retroperitoneal myoma	TAH with BSO	Bilateral/proximal *	No	Therapeutic
3	35	No	Uterine myoma	Myomectomy	Bilateral/proximal *	No	Therapeutic
4	44	AHT class I	Uterine myoma	TAH without BSO	Bilateral/proximal *	No	Therapeutic
5	46	No	Uterine myoma	TAH with BSO	Unilateral/proximal	Lesion of internal iliac vein	Therapeutic
6	49	Varicosis cruris	Uterine myoma/ Ovarian cyst	TAH with BSO	Bilateral/proximal **	Pulmonary embolism	Prophylactic
7	44	DM type II, AHT class III, LSHF class III, aortic prosthesis	Uterine myoma	TAH with BSO	Bilateral/proximal **	No	Therapeutic
8	50	Anemia	Retroperitoneal myoma	TAH with BSO	Bilateral/proximal	No	Prophylactic
9	51	AHT class II	Retroperitoneal myoma	TAH with BSO	Bilateral/proximal	No	Prophylactic
10	71	AHT class II	Ovarian cyst	TAH with BSO	Unilateral/proximal	No	Therapeutic
11	62	AHT class II	Ovarian cyst	TAH with BSO	Unilateral/proximal *	No	Therapeutic
12	76	AHT class II	PID, unilateral parametritis	TAH with BSO	Unilateral/proximal	No	Therapeutic
13	54	AHT class II	Vaginal vault prolapse	LSCP	Bilateral/proximal **	No	Therapeutic

* Ligation before the main procedure; ** Ligation after relaparotomy for postoperative bleeding. AHT—arterial hypertension; TAH—total abdominal hysterectomy; BSO—bilateral salpingo-oophorectomy; LSCP—laparoscopic sacrocolpopexy; DM—diabetes mellitus; LSHF—left-sided heart failure; PID—pelvic inflammatory disease.

**Table 4:** History of patients who underwent IIA ligation in gynecologic oncology group.

No	Age	Comorbidities	Diagnosis	Procedure	Type of Ligation	Intra- and Postoperative Complications	Type of Procedure
1	44	No	Cervical cancer	RH with BSO/ PLND	Bilateral/proximal	Uretero- vaginal fistula	Therapeutic
2	56	Hashimoto thyroiditis, DM type II	Cervical cancer	RH with BSO/ PLND	Bilateral/proximal	No	Therapeutic
3	67	Parkinson’s disease	Cervical cancer	RH with BSO/ PLND	Bilateral/distal	No	Therapeutic
4	57	Multiple myeloma, AHT class III, Thalassemia minor	Cervical cancer	RH with BSO/ PLND	Bilateral/ Left -distal, right- proximal	No	Therapeutic
5	54	AHT class II	Cervical cancer	RH with BSO/ PLND	Bilateral/ distal	Uretero-vaginal fistula	Therapeutic
6	66	AHT class II	Cervical cancer	RH with BSO/ PLND	Bilateral/ proximal	No	Therapeutic
7	50	No	Cervical cancer	RH with BSO/ PLND	Bilateral/ proximal	Uretero-vaginal fistula	Therapeutic
8	55 *	AHT class II	Cervical cancer	TH with BSO/PLND *	Bilateral/proximal Abdominal packing	Lesion of internal iliac vein during PLND	Therapeutic
9	76	AHT class II	Cervical cancer	RH with BSO/PLND	Bilateral/proximal	No	Therapeutic
10	64	AHT class II	Cervical cancer	RH with BSO/ PLND	Bilateral/proximal	No	Therapeutic
11	61	AHT class II, DM type II	Cervical cancer- CS	RH with BSO/ PLND	Bilateral/proximal	No	Therapeutic
12	54	Anemia	Advanced cervical cancer	Extraperitoneal ligation of the IIA	Bilateral/proximal	Persistent bleeding	Palliative
13	47	Anemia	Advanced cervical cancer	Extraperitoneal ligation of the IIA	Bilateral/proximal	Persistent bleeding	Palliative
14	49	Anemia	Advanced cervical cancer	Extraperitoneal ligation of the IIA	Unilateral/proximal	Persistent bleeding	Palliative
15	53	AHT class III, Anemia	ULMS	TH with BSO	Bilateral/ proximal **	No	Therapeutic
16	44	Anemia	UUS	TH with BSO/ PLND/PALND ***	Bilateral/ proximal **	No	Therapeutic
17	73	AHT class II	ULMS	TH with BSO	Unilateral/ proximal	No	Prophylactic
18	65	AHT class III, DM type II, IHD	ULMS	TH with BSO	Bilateral/ proximal **	No	Therapeutic
19	66	AHT class II, COPD	ULMS	TH with BSO	Unilateral/ proximal	No	Prophylactic
20	78	AHT class III	HGESS	TH with BSO/PLND ***	Bilateral/proximal **	No	Therapeutic
21	57	AHT class II	EC	TH with BSO/PLND	Bilateral/proximal	No	Therapeutic
22	67	Hashimoto’s thyroiditis	ECS	TH with BSO/PLND/RSR	Bilateral/ proximal	No	Therapeutic
23	64	No	OC	Hudson procedure/TO/DS/	Bilateral/proximal ****	No	Therapeutic
24	77	AHT class III, IHD, COPD	OC	Hudson’s procedure/TO/DS	Bilateral/proximal	Persistent bleeding–conservatively managed	Therapeutic
25	31	No	OC	Th with BSO/TO	Bilateral/left- distal, right- proximal	No	Prophylactic
26	72	AHT class II	OC	TH with BSO/TO	Bilateral /proximal	No	Therapeutic
27	76	AHT class II, DM type II	OC	Hudson procedure/TO	Bilateral/proximal	No	Therapeutic
28	79	AHT class III	OC	Hudson procedure/TO	Bilateral/proximal	No	Therapeutic
29	79	AHT class III, HF class II,	OC	Hudson procedure/TO	Bilateral/left- proximal, right–distal	No	Therapeutic
30	81	AHT class III, DM type II	VC	RH/PLND	Bilateral/ proximal	No	Therapeutic

* Patient after primary chemoradiation (intended as definitive treatment); the operation was performed due to residual cervical tumor. ** The artery was ligated before hysterectomy due to massive intraoperative bleeding *** Pelvic and paraaortic lymph node dissection was performed due to bulky lymph nodes as part of optimal cytoreduction in patients with uterine sarcomas; **** The artery was ligated during relaparotomy for postoperative bleeding; AHT—arterial hypertension; DM—diabetes mellitus; COPD—chronic obstructive pulmonary disease; IHD—ischemic heart disease; ULMS—uterine leiomyosarcoma; UUS—undifferentiated uterine sarcoma; HGESS—high-grade endometrial stromal sarcoma; RH—radical hysterectomy; BSO—bilateral saplingo-ophorectomy; TO—total omentectomy; PLND—pelvic lymph node dissection; HF—heart failure; DS—diaphragmatic stripping; PALND—paraaortic lymph node dissection.

## Data Availability

Data are available on reasonable request from the first author.

## Appendix A

**Table A1:** Hemoglobin Levels, Transfusions, and Reinterventions—Obstetric Cases.

Pat. No.	Preop Hb (g/dL)	Postop Hb (g/dL)	Hb Drop (g/dL)	Total PRBC/FFP Units per Patient	Reintervention
1	8.0	7.9	0.1	2/2	No
2	11.5	6.1	5.4	4/4	No *
3	11.5	11.0	0.5	0	No
4	10.0	9.1	0.9	2/2	No
5	10.0	10.9	−0.9	1/1	No
6	9.3	11.0	−1.7	1/1	No
7	9.8	10.2	−0.4	1/1	No
8	9.2	9.8	−0.6	2/2	No
9	9.4	10.7	−1.3	4/3	No
10	7.7	7.1	0.6	2/2	No
11	10.9	9.0	1.9	0	No
12	7.8	6.2	1.6	4/4	No
13	12.8	8.1	4.7	3/3	No *
14	10.8	8.1	2.7	1/1	No
15	10.5	8.9	1.6	1/1	No
16	8.5	9.1	−0.6	2/2	No
17	11.4	7.4	4.0	3/3	No
18	11.0	7.7	3.3	3/3	No

* Damage-control surgery with packing/laparostomy and delayed abdominal closure; no reintervention for recurrent bleeding after IIA ligation.

**Table A2:** Hemoglobin Levels, Transfusions, and Reinterventions—Benign Gynecology Cases.

Pat. No.	Preop Hb (g/dL)	Postop Hb (g/dL)	Hb Drop (g/dL)	Total PRBC/FFP Units per Patient	Reintervention
1	9.8	8.5	1.3	1/1	No
2	8.2	6.1	2.1	2/2	No
3	12.8	7.4	5.4	2/2	No
4	16.7	13.5	3.2	0/0	No
5	11.1	10.0	1.1	0/0	No
6	13.3	7.5	5.8	2/2	No *, **
7	9.2	7.3	1.9	2/2	No *
8	11.0	9.0	2.0	0/0	No
9	14.0	8.8	5.2	1/1	No
10	13.3	11.7	1.6	0/0	No
11	14.2	11.9	2.3	0/0	No
12	9.6	11.1	−1.5	1/0	No
13	12.6	10.7	1.9	1/1	No *

* The IIA ligation was performed during a re-laparotomy due to postoperative bleeding, ** The first postoperative period (before re-laparotomy with IIA ligation) complicated by pulmonary embolism.

**Table A3:** Hemoglobin Levels, Transfusions, and Reinterventions—Gynecologic Oncology Cases.

Pat. No.	Preop Hb (g/dL)	Postop Hb (g/dL)	Hb Drop (g/dL)	Total PRBC/FFP Units per Patient	Reintervention
1	9.6	7.8	1.8	0/0	No
2	13.6	8.7	4.9	0/0	No
3	12.9	10.0	2.9	0/0	No
4	9.4	8.9	0.5	0/0	No
5	15.3	12.2	3.1	0/0	No
6	11.7	9.0	2.7	0/0	No
7	14.3	10.7	3.6	0/0	No
8	14.2	7.6	6.6	3/3	No
9	13.0	10.0	3.0	0/0	No
10	12.0	9.1	2.9	0/0	No
11	11.9	9.3	2.6	0/0	No
12	7.0	7.7	−0.7	2/2	No
13	9.0	7.7	1.3	2/2	No
14	7.5	7.0	0.5	2/2	No
15	4.0	8.7	−4.7	4/3	No
16	7.6	8.1	−0.5	4/4	No
17	11.1	8.6	2.5	1/1	No
18	10.0	8.8	1.2	1/1	No
19	11.3	7.9	3.4	2/2	No
20	13.6	9.3	4.3	2/2	No
21	15.5	13.0	2.5	0/0	No
22	8.4	7.5	0.9	2/1	No
23	13.4	7.1	6.3	2/1	No
24	12.1	10.4	1.7	0/0	No
25	11.9	9.2	2.7	0/0	No
26	10.2	9.0	1.2	0/0	No
27	8.1	8.7	−0.6	3/0	No
28	11.0	9.0	2.0	0/0	No
29	10.9	10.3	0.6	0/0	No
30	14.2	11.3	2.9	0/0	No
